# IFNL4 ss469415590 Variant Is Associated with Treatment Response in Japanese HCV Genotype 1 Infected Individuals Treated with IFN-Including Regimens

**DOI:** 10.1155/2014/723868

**Published:** 2014-12-08

**Authors:** Tatsuo Miyamura, Tatsuo Kanda, Shingo Nakamoto, Makoto Arai, Masato Nakamura, Shuang Wu, Xia Jiang, Reina Sasaki, Yuki Haga, Shin Yasui, Yoshihiko Ooka, Tetsuhiro Chiba, Fumio Imazeki, Shigeru Mikami, Osamu Yokosuka

**Affiliations:** ^1^Departments of Gastroenterology and Nephrology, Graduate School of Medicine, Chiba University, 1-8-1 Inohana, Chuo-ku, Chiba 260-8677, Japan; ^2^Department of Molecular Virology, Graduate School of Medicine, Chiba University, Chiba 260-8677, Japan; ^3^Department of Gastroenterology, Kikkoman General Hospital, Noda 278-0005, Japan

## Abstract

*Aim*. Eradication of hepatitis C virus (HCV) is still challenging even if interferon- (IFN-) free regimens with direct-acting antiviral agents (DAAs) for HCV-infected individuals are available in clinical practice. IFNL4 is a newly described protein, associated with human antiviral defenses. We investigated whether IFNL4 ss469415590 variant has an effect on the prediction of treatment response in HCV-infected patients treated with IFN-including regimens. *Patients and Methods*. In all, 185 patients infected with HCV genotype 1 treated with peg-IFN plus ribavirin, with or without telaprevir, were genotyped for IFNL4 ss469415590. We retrospectively investigated whether the role of IFNL4 ss469415590 variant and other factors could predict sustained virological response (SVR) in Japanese patients infected with HCV genotype 1. *Results*. There were 65.7%, 31.5%, and 2.8% patients in the IFNL4 ss469415590 TT/TT, TT/-G, and -G/-G groups, respectively. SVR rates were 82.1% or 49.3% in patients treated with peg-IFN plus ribavirin with or without telaprevir, respectively. IFNL4 ss469415590 variant and HCV viral loads or IFNL4 ss469415590 variant and early virological response were better predictors of SVR in patients treated with peg-IFN plus ribavirin with or without telaprevir, respectively. *Conclusion*. In the era of DAAs, measurement of IFNL4 ss469415590 variant could help the prediction of SVR in Japanese HCV genotype 1 infected individuals treated with IFN-including regimens.

## 1. Introduction

Chronic hepatitis C virus (HCV) infection is a leading cause of hepatocellular carcinoma (HCC) and end-stage liver disease requiring liver transplantation in the United States [1, 2]. In Japan, HCV infection is still a major cause of HCC [3]. At present, HCV infection is a world health problem.

The former standard treatment with peginterferon (peg-IFN) plus ribavirin led to ~50% and ~80% sustained virological response (SVR) rates in patients infected with HCV genotypes 1 and 2, respectively [4]. Since 2011, peg-IFN plus ribavirin with telaprevir or boceprevir has been available in the United States as well as that with telaprevir in Japan. These treatments resulted in higher SVR rates (~70% and ~80% for treatment-naïve patients and previous treatment relapsers, resp.) in HCV genotype 1 infected patients [5].

Genome-wide association studies (GWAS) [6–8] revealed that several single nucleotide polymorphisms (SNPs) near the IFNL3 (previously called IL28B) coding region, such as rs8099917 and rs12978690, are associated with treatment response to peg-IFN plus ribavirin treatment. We also reported that IFNL3 rs8099917 was useful for the prediction of SVR in patients infected with HCV genotype 1 [9]. However, the mechanism between IFNL3 and clearance of HCV has been unclear despite the extensive work [10–12]. We also reported that IFNL3 induces IFN-stimulated genes (ISGs) such as IFN-*γ*-inducible protein (IP-10) that are reportedly associated with the progression of HCV-related pathogenesis and antiviral activities against HCV [12].

A recent study [13] showed that a variant upstream of IFNL3 could create IFNL4, impairing the clearance of HCV. Bibert et al. [14] reported that induction of IL28B and IP-10 mRNA relies on IFNL4 ss469415590 TT/-G variant in peripheral blood mononuclear cells (PBMCs), and this variant would improve the prediction of treatment response to peg-IFN and ribavirin [14, 15].

So far, only a few studies are available about the association between IFNL4 ss469415590 variant and treatment response in Japanese patients infected with HCV genotype 1. Peg-IFN plus ribavirin with telaprevir is also still one of the standard care treatments for chronic hepatitis C genotype 1 patients, at least until IFN-free regimens become generally available in clinical daily practice.

In the present study, the usefulness of IFNL4 ss469415590 variant for the prediction of virological response in chronic hepatitis C patients treated with peg-IFN plus ribavirin with or without telaprevir was investigated.

## 2. Patients and Methods

### 2.1. Patients

The present study comprised a total of 185 patients with chronic hepatitis C treated with peg-IFN plus ribavirin with or without telaprevir at the Department of Gastroenterology, Chiba University Hospital, and the Department of Gastroenterology, Kikkoman General Hospital in Chiba, Japan. Of these patients, 81 patients treated with peg-IFN plus ribavirin had been already included in previous reports [9, 11, 16]. Written informed consent was obtained from all patients, and the study was approved by the Ethics Committee of Chiba University, School of Medicine (number 428). All patients were of Asian origin and infected with HCV genotype 1. They underwent standard antiviral therapy with the combination of peg-IFN plus ribavirin [4] or telaprevir with peg-IFN plus ribavirin [17, 18]. HCV RNA was measured at baseline, at weeks 4 and 12, at the end of treatment, and at 24 weeks after the end of treatment. SVR was defined as undetectable serum HCV RNA at 24 weeks after the end of treatment. Patients with undetectable HCV RNA at 4 and 12 weeks after the commencement of treatment were considered to have had rapid virological response (RVR) and early virological response (EVR), respectively [19]. Relapse was defined as undetectable HCV RNA at the end of treatment, but it reappeared after the end of treatment [20]. Null response was defined as less than 2 log_10_ decrease in HCV RNA (IU/mL) from baseline after 12 weeks of therapy [20].

### 2.2. DNA Extraction and IFNL4 Variant Genotyping

Blood DNA was extracted by DNA extracted all lysis reagents (Applied Biosystems, Foster City, CA, USA). IFNL4 ss469415590 was determined by TaqMan SNP assay. Primers were purchased from Applied Biosystems. Thermal cycling was performed with the ABI Step One real-time PCR system as previously described [9]. We analyzed IFNL4 ss469415590 TT/TT as major genotype and TT/-G and -G/-G as minor genotypes in the present study.

### 2.3. Measurement of HCV RNA in Serum

HCV RNA was measured by COBAS AMPLICOR HCV Monitor test v. 2.0 (range: 0.5–850 KIU/mL, lower limit of detection: 1.7 log IU/mL) or COBAS TaqMan HCV test with levels ranging from 1.2 to 7.8 log IU/mL (Roche Diagnostics, Tokyo, Japan). In all patients treated with telaprevir with peg-IFN plus ribavirin, HCV RNA was measured by COBAS TaqMan HCV test.

### 2.4. Statistical Analysis

Data are expressed as mean ± SD. Variables with categorical data were compared among groups using chi-square test and Student's *t*-test as indicated. *P* < 0.05 was considered statistically significant. Variables with *P* < 0.1 at univariate analysis were retained for multivariate logistic regression analysis. Statistical analysis was performed using the Excel statistics program for Windows, version 7 (SSRI, Tokyo, Japan).

## 3. Results

IFNL4 ss469415590 variant was determined in 178 of the total 185 patients, and 117 (65.7%) had TT/TT, 56 (31.5%) had TT/-G, and 5 (2.8%) had -G/-G of ss469415590 in the present study (Table 1). In 141 patients treated with peg-IFN plus ribavirin without telaprevir, 93 (66.0%) had TT/TT, 43 (30.5%) had TT/-G, and 5 (3.5%) had -G/-G of ss469415590 (Table 2). In 37 patients treated with peg-IFN, ribavirin, and telaprevir, 24 (64.9%) had TT/TT and 13 (35.1%) had TT/-G of ss469415590 (Table 3). The distribution of IFNL4 ss469415590 variant of both treatment groups was quite similar.

In 24-week follow-up after treatment, 82.1% or 49.3% of patients treated with peg-IFN plus ribavirin with or without telaprevir, respectively, achieved SVR (Table 1). To clarify the predictors of SVR, we compared the baseline characteristics and treatment response between SVR and non-SVR groups of the patients treated with peg-IFN plus ribavirin (Table 4(a)). Univariate analysis showed that previous treatment response, *γ*-GTP levels, and platelet count, having RVR, EVR, and IFNL4 ss469415590 variant in the patients treated with peg-IFN plus ribavirin, contributed to the achievement of SVR. Factors associated with SVR by univariate analysis were then subjected to multivariate logistic regression analysis. SVR was attained independently of IFNL4 ss469415590 major genotype and having EVR in these patients treated with peg-IFN plus ribavirin (Table 4(b)).

Next, we compared the baseline characteristics and treatment response between SVR and non-SVR groups of the patients treated with peg-IFN, ribavirin, and telaprevir (Table 5(a)). HCV RNA at baseline was included, as the measurement of HCV RNA detection was uniform in this group (see Section 2). Univariate analysis showed that having RVR and EVR in the patients treated with peg-IFN, ribavirin, and telaprevir contributed to achievement of SVR. Factors associated with SVR by univariate analysis were then subjected to multivariate logistic regression analysis. SVR was attained independently of lower HCV RNA at baseline, and IFNL4 ss469415590 major genotype in these patients was treated with peg-IFN plus ribavirin and telaprevir (Table 5(b)).

## 4. Discussion

The present study confirmed previous studies that IFNL4 ss469415590 variant plays an important role in eradicating HCV genotype 1 from patients treated with peg-IFN plus ribavirin with or without telaprevir [14, 21–23]. Multivariate analysis revealed that IFNL4 ss469415590 major genotype was statistically associated with achieving SVR in patients treated with peg-IFN plus ribavirin as well as those treated with peg-IFN, ribavirin, and telaprevir.

IFNL3-associated SNPs were strongly associated with treatment response to peg-IFN plus ribavirin without telaprevir treatment [6–9]. Although there have been several reports on the mechanism involving IFNL3 [10, 11], the exact mechanism or function of IFNL3 has yet to be clarified. Bibert et al. [14] reported that induction of IL28B and IP-10 mRNA relied on ss469415590 TT/-G but not rs12979860, making ss469415590 TT/-G the only functional variant identified, although there are contrary opinions [24, 25].

Prokunina-Olsson et al. [13] found that several novel transcripts and ss469415590 are specific for the region upstream of IFNL3. Their global protein BLAST search [13] showed that 179 amino acids (p179) from transcripts with the ss469415590-G allele have only homology with 29.1% amino acid identity and 40.8% amino acid similarity with IFNL3 among predictive proteins from the 10 novel transcripts. IFNL3 and these p179 (IFNL4) proteins are most related within the sequences, corresponding to the A and F helices of IFNL3, which constitute the core area for interaction of IFNL3 with its primary receptor, IFNLR1 (IL28R1) [13]. The beneficial insertion ss469415590-TT allele could abrogate IFNL4. IFNL4 signaling via IFN*λ* receptor could regulate antiviral activity against coronaviruses as well as HCV [26]. Manuel et al. [27] also reported that polymorphisms in the IFNL3/4 region influence susceptibility to CMV replication in solid-organ transplant recipients, suggesting that IFNL4 as well as IFNL3 play important roles in human antiviral defenses.

Our results indicated that IFNL4 ss469415590 variant is useful for prediction of the treatment response to IFN-including regimens. A recent study [28] showed that IFNL4 ss469415590 minor genotype was associated with slower early viral decay, due to slower loss of free virus and decreased drug efficacy in patients treated with the DAA sofosbuvir against HCV NS5B, along with RBV, suggesting that IFNL4 variant might be useful in the DAA era. In IFN-free DAA therapies, it is unclear whether these variants play a role or not, although it is possible that certain DAAs could have effects on IFN signaling such as ISGs of host cells.

## 5. Conclusions

Measurement of IFNL4 ss469415590 variant is another option for prediction of the treatment response to IFN-including regimens in Japanese HCV genotype 1 infected individuals. Together, the mechanism of the interaction between treatment response and IFNL4 as well as IFNL3 in HCV-infected patients should be further explored.

## Figures and Tables

**Table 1:** Baseline characteristics of patients in the present study.

	All patients, *n* = 185	Patients with dual therapy, *n* = 146	Patients with triple therapy, *n* = 39	*P* values (dual therapy versus triple therapy groups)
Gender, male/female	102/83	73/73	29/10	0.011
Age, years	56.2 ± 10.7	55.7 ± 11.2	58.2 ± 8.7	0.197
Previous treatment: naïve/relapse/null	119/45/21	102/26/18	17/19/3	0.0043 (naïve versus non-naïve)
AST, IU/L	57.1 ± 48.0	58.0 ± 48.7	53.9 ± 45.9	0.630
ALT, IU/L	70.4 ± 64.7	71.0 ± 64.9	68.1 ± 64.8	0.800
*γ*-GTP, IU/L	57.0 ± 66.7	56.4 ± 68.6	59.2 ± 59.8	0.816
Hemoglobin, g/dL	14.1 ± 1.3	14.0 ± 1.2	14.6 ± 1.3	0.0070
Platelets, ×10^4^/mm^3^	16.6 ± 5.6	16.8 ± 5.8	16.0 ± 5.1	0.434
Treatment response				
RVR, *n*	42	15	27	<0.0001
EVR, *n*	99	65	34	<0.0001
SVR/relapse/null	104/39/42	72/37/37	32/2/5	<0.0001
IFNL4, ss469415590, major/minor/ND	117/61/7	93/48/5	24/13/2	0.944

Peginterferon plus ribavirin with or without telaprevir therapy was defined as triple or dual therapy, respectively. Null: null response; AST: aspirate aminotransferase; ALT: alanine aminotransferase; *γ*-GTP: *γ*-glutamyltransferase; RVR: rapid virological response; EVR: early virological response; SVR: sustained virological response.

**Table 2:** Baseline characteristics and treatment response in 141 patients treated with peginterferon plus ribavirin according to IFNL4 ss469415590 variant.

	IFNL4 major, *n* = 93	IFNL4 minor, *n* = 48	*P* values (major versus minor groups)
Gender, male/female	46/47	25/23	0.900
Age, years	56.1 ± 11.7	54.7 ± 10.6	0.488
Previous treatment: naïve/relapse/null	68/19/6	29/7/12	0.0042
AST, IU/L	53.4 ± 48.5	65.0 ± 44.0	0.167
ALT, IU/L	65.1 ± 64.9	82.0 ± 63.3	0.14
*γ*-GTP, IU/L	40.9 ± 39.2	87.5 ± 98.9	0.000107
Hemoglobin, g/dL	13.9 ± 1.2	14.2 ± 1.2	0.16
Platelets, ×10^4^/mm^3^	16.8 ± 5.7	16.8 ± 6.1	1.00
Treatment response			
RVR, *n*	15	0	0.0079
EVR, *n*	59	5	<0.0001
SVR/relapse/null	60/23/10	10/12/25	<0.0001

Null: null response; AST: aspirate aminotransferase; ALT: alanine aminotransferase; *γ*-GTP: *γ*-glutamyltransferase; RVR: rapid virological response; EVR: early virological response; SVR: sustained virological response.

**Table 3:** Baseline characteristics and treatment response in 37 patients treated with peginterferon, ribavirin, and telaprevir according to IFNL4 ss469415590 variant.

	IFNL4 major, *n* = 24	IFNL4 minor, *n* = 13	*P* values (major versus minor groups)
Gender, male/female	19/5	9/4	0.78
Age, years	59.5 ± 7.7	55.2 ± 10.2	0.15
Previous treatment: naïve/relapse/null	10/13/1	7/4/2	0.71
AST, IU/L	50.9 ± 48.9	59.8 ± 44.2	0.58
ALT, IU/L	63.8 ± 60.5	77.9 ± 77.2	0.54
*γ*-GTP, IU/L	51.8 ± 52.2	78.6 ± 73.3	0.25
Hemoglobin, g/dL	14.6 ± 1.2	14.5 ± 1.3	0.81
Platelets, ×10^4^/mm^3^	15.9 ± 5.2	16.5 ± 5.3	0.74
HCV RNA (log⁡IU/mL)	6.70 ± 0.54	6.18 ± 0.87	0.0309
Treatment response			
RVR, *n*	17	8	0.83
EVR, *n*	22	10	0.45
SVR/relapse/null	22	8	0.0727

Null: null response; AST: aspirate aminotransferase; ALT: alanine aminotransferase; *γ*-GTP: *γ*-glutamyltransferase; RVR: rapid virological response; EVR: early virological response; SVR: sustained virological response.

**Table:** (a) Univariate analysis

	SVR, *n* = 72	Non-SVR, *n* = 74	*P* values (SVR versus non-SVR groups)
Gender, male/female	39/33	34/40	0.40
Age, years	54.4 ± 12.7	57.0 ± 9.3	0.15
Previous treatment: naïve/relapse/null	59/12/1	43/14/17	0.00020
AST, IU/L	54.1 ± 54.3	61.8 ± 42.5	0.34
ALT, IU/L	69.0 ± 71.5	73.0 ± 58.2	0.71
*γ*-GTP, IU/L	42.7 ± 41.3	70.0 ± 85.7	0.0158
Hemoglobin, g/dL	13.9 ± 1.1	14.1 ± 1.3	0.31
Platelets, ×10^4^/mm^3^	17.8 ± 5.4	15.7 ± 5.9	0.0265
Treatment response			
RVR, *n*	15	0	0.000107
EVR, *n*	53	12	<0.0001
IFNL4, ss469415590, major/minor/ND	60/10/2	33/38/3	<0.0001

Null: null response; AST: aspirate aminotransferase; ALT: alanine aminotransferase; *γ*-GTP: *γ*-glutamyltransferase; RVR: rapid virological response; EVR: early virological response; SVR: sustained virological response.

**Table:** (b) Factors associated with SVR among patients treated with peginterferon plus ribavirin by multivariate analysis

Factor	Category	Odds ratio	95% CI	*P* values
IFNL4, ss469415590	major/minor	2.56	1.005–6.557	0.0486
EVR	+/−	10.16	4.28–24.10	<0.0001

EVR: early virological response.

**Table:** (a) Univariate analysis

	SVR, *n* = 32	Non-SVR, *n* = 7	*P* values (SVR versus non-SVR groups)
Gender, male/female	24/8	5/2	0.77
Age, years	57.8 ± 9.3	59.8 ± 5.6	0.58
Previous treatment: naïve/relapse/null	14/17/1	3/2/2	0.13
AST, IU/L	55.9 ± 50.1	44.7 ± 16.0	0.56
ALT, IU/L	71.9 ± 70.6	51.1 ± 19.9	0.44
*γ*-GTP, IU/L	62.1 ± 64.7	46.2 ± 27.9	0.53
Hemoglobin, g/dL	14.6 ± 1.3	14.4 ± 1.6	0.72
Platelets, ×10^4^/mm^3^	16.4 ± 5.2	14.3 ± 4.6	0.33
HCV RNA (log⁡IU/mL)	6.45 ± 0.68	6.81 ± 0.75	0.220
Treatment response			
RVR, *n*	27	0	<0.0001
EVR, *n*	32	2	<0.0001
IFNL4, major/minor/ND	22/8/2	2/5/0	0.0727

Null: null response; AST: aspirate aminotransferase; ALT: alanine aminotransferase; *γ*-GTP: *γ*-glutamyltransferase; RVR: rapid virological response; EVR: early virological response; SVR: sustained virological response.

**Table:** (b) Factors associated with SVR among patients treated with peginterferon, ribavirin, and telaprevir by multivariate analysis

Factor	Category	Odds ratio	95% CI	*P* values
HCV RNA (log IU/mL)	<6.4/6.4≤	13.74	1.014–186.30	0.048
IFNL4 ss469415590	major/minor	17.54	1.980–156.25	0.010
